# Recurrence patterns and progression-free survival after chemoradiotherapy with or without consolidation durvalumab for stage III non-small cell lung cancer

**DOI:** 10.1093/jrr/rrac057

**Published:** 2022-09-22

**Authors:** Noriko Kishi, Yukinori Matsuo, Takashi Shintani, Masakazu Ogura, Takamasa Mitsuyoshi, Norio Araki, Kota Fujii, Setsuko Okumura, Kiyoshi Nakamatsu, Takahiro Kishi, Tomoko Atsuta, Takashi Sakamoto, Shuji Ohtsu, Tomohiro Katagiri, Masaru Narabayashi, Satsuki Fujishiro, Yusuke Iizuka, Hiroaki Ozasa, Toyohiro Hirai, Takashi Mizowaki, Kyoto Radiation Oncology Study Group (KROSG)

**Affiliations:** Department of Radiation Oncology and Image-Applied Therapy, Graduate School of Medicine, Kyoto University, 54 Shogoin-Kawahara-cho, Sakyo-ku, Kyoto, 606-8507, Japan; Department of Radiation Oncology and Image-Applied Therapy, Graduate School of Medicine, Kyoto University, 54 Shogoin-Kawahara-cho, Sakyo-ku, Kyoto, 606-8507, Japan; Department of Radiology, Japanese Red Cross Fukui Hospital, 2-4-1 Tsukimi, Fukui, 918-8501, Japan; Department of Radiation Oncology, Kishiwada City Hospital, 1001 Gakuhara-cho, Kishiwada, Osaka, 596-8501, Japan; Department of Radiation Oncology, Kobe City Medical Center General Hospital, 2-1-1, Minatojimaminamimachi, Chuo-ku, Kobe, Hyogo, 650-0047, Japan; Department of Radiology, National Hospital Organization Kyoto Medical Center, 1-1 Fukakusamukaihata-cho, Fushimi-ku, Kyoto, 612-8555, Japan; Department of Radiation Oncology, Kurashiki Central Hospital, 1-1-1 Miwa, Kurashiki, Okayama, 710-8602, Japan; Department of Radiation Oncology, Hyogo Prefectural Amagasaki General Medical Center, 2-17-77 Higashinanba-cho, Amagasaki, Hyogo, 660-8550, Japan; Department of Radiation Oncology, Kindai University Faculty of Medicine, 377-2, Onohigashi, Osakasayama-shi, Osaka, 589-8511, Japan; Department of Radiation Oncology, Osaka Red Cross Hospital, 5-30 Fudegasaki-cho, Tennoji-ku, Osaka, 543-8555, Japan; Department of Radiation Oncology, Kitano Hospital, Tazuke Kofukai Medical Research Institute, 2-4-20, Ohgimachi, Kita-ku, Osaka, 530-8480, Japan; Department of Radiation Oncology, Kyoto Katsura Hospital, 17 Yamadahirao-cho, Nishikyo-ku, Kyoto, 615-8256, Japan; Department of Radiation Oncology, Kyoto City Hospital, 1-2 Mibuhigashitakada-cho, Nakagyo-ku, Kyoto, 604-8845, Japan; Department of Radiation Oncology, Tenri Hospital, 200 Mishima-cho, Tenri, Nara, 632-8552, Japan; Department of Radiology, Japanese Red Cross Fukui Hospital, 2-4-1 Tsukimi, Fukui, 918-8501, Japan; Department of Radiation Oncology, Shinko Hospital, 1-4-47 Wakihama-cho, Chuo-ku, Kobe, Hyogo, 651-0072, Japan; Department of Radiation Oncology, Shizuoka City Shizuoka Hospital, 10-93 Otemachi, Aoi-ku, Shizuoka, 420-8630, Japan; Department of Respiratory Medicine, Graduate School of Medicine, Kyoto University, 54 Shogoin-Kawahara-cho, Sakyo-ku, Kyoto, 606-8507, Japan; Department of Respiratory Medicine, Graduate School of Medicine, Kyoto University, 54 Shogoin-Kawahara-cho, Sakyo-ku, Kyoto, 606-8507, Japan; Department of Radiation Oncology and Image-Applied Therapy, Graduate School of Medicine, Kyoto University, 54 Shogoin-Kawahara-cho, Sakyo-ku, Kyoto, 606-8507, Japan

**Keywords:** locoregional recurrence (LR), distant metastasis, real-world data, immunotherapy

## Abstract

Chemoradiotherapy followed by consolidation durvalumab (CCRT+D) improves survival in patients with stage III non-small-cell lung cancer (NSCLC). We compared recurrence patterns and survival in the CCRT+D and CCRT cohorts. We conducted a multicenter, retrospective study in Japan. Patients who received CCRT for stage III NSCLC were included in this study. Of 178 eligible patients, 136 were in the CCRT+D and 42 were in the CCRT cohorts. Locoregional recurrence (LR), LR plus distant metastases (DM), and DM were observed in 20.6%, 8.8%, 27.9% of the CCRT+D, and 26.2%, 16.7% and 33.3% of the CCRT cohorts, respectively. In-field recurrence was the most common LR pattern in both cohorts. Squamous cell carcinoma and PD-L1 expression < 1%, and female sex and EGFR mutations were significantly associated with an increased risk of LR and DM. In patients with any risk factors for LR, the incidence of LR was similar in the CCRT+D and CCRT (39.5% vs 45.5%). The 24 month progression-free survival (PFS) and overall survival (OS) were 40.3% and 69.4% in the CCRT+D and 24.7% and 61.0% in the CCRT cohorts, respectively. Poor performance status and no consolidation durvalumab were significantly associated with shorter PFS. There was a significant difference in PFS between the CCRT+D and CCRT in the propensity score-matched cohort (HR = 0.51, *P* = 0.005). **In conclusion,** consolidation durvalumab decreased both LR and DM, and significantly improved PFS. However, in-field recurrence was still a major problem, as well as DM.

## INTRODUCTION

Non-small cell lung cancer (NSCLC) accounts for more than 80% of lung cancers, and locally advanced NSCLC accounts for approximately 35% of NSCLC cases [1]. The standard treatment strategy for locally advanced NSCLC has long been concurrent chemoradiotherapy (CCRT) [2] and has not changed drastically over the years. The PACIFIC trial, a phase 3 study that compared the anti-programmed death-ligand 1 (PD-L1) antibody durvalumab with placebo as consolidation therapy after CCRT, is a practice-changing study as it demonstrated that consolidation durvalumab significantly improved overall survival (OS) in patients with unresectable stage III NSCLC [3]. According to the updated study analysis, the 5 year OS rate was 42.9% for the durvalumab group, superior to 33.4% for the placebo group [4].

Two points remain unclear in the PACIFIC trial. First, information on in-field recurrence was unavailable. The most common sites of new lesions (24.2% of the durvalumab group and 33.3% of the placebo group) were the lungs, lymph nodes and brain [4], but it is unclear whether the new lesions in the lungs or lymph nodes were within the irradiated field. In-field recurrence is a negative prognostic factor for survival after CCRT for inoperable stage III NSCLC [5], which causes fatal symptoms such as bleeding or compression of the trachea, bronchus, or superior vena cava. From the radiation oncologist’s perspective, it is critical to understand the detailed patterns and risk factors of local recurrence, which might lead to further improvement of the outcomes by refining the radiotherapy dose and techniques. Second, the efficacy of consolidation durvalumab in real-world settings is unknown. There might be a selection bias in the randomized controlled trial, and patients with disease progression after CCRT, unresolved toxicities grade 2 or worse, or deteriorated general condition have not been included in the PACIFIC trial. Real-world data, including these patients compared to the historical cohort, have been reported [6–8]. However, this comparison with historical data is subject to another selection bias because the OS of unresectable stage III NSCLC has improved significantly over time [9]. Therefore, comparing outcomes between patients with or without consolidation durvalumab in the same period would elucidate its efficacy in a real-world setting.

We conducted a multicenter retrospective study to evaluate the incidence and risk factors of symptomatic radiation pneumonitis in NSCLC treated with CCRT and consolidation durvalumab at 12 months of follow-up [10]. After a 24 month follow-up, the same study cohort was used to collect data on survival and detailed patterns of recurrence in relation to the dose distribution of radiotherapy in patients with NSCLC who received CCRT plus consolidation durvalumab, as well as in patients with NSCLC who received CCRT without durvalumab. This study aimed to evaluate the detailed patterns and risk factors for recurrence considering the involved field, and compare CCRT outcomes with consolidation durvalumab and CCRT outcomes without consolidation durvalumab in patients with stage III NSCLC.

## PATIENTS AND METHODS

This multi-institutional retrospective observational study was performed in accordance with the Declaration of Helsinki (1975; revised in 2013). Fifteen Japanese institutions of the Kyoto Radiation Oncology Study Group (KROSG) participated in this study after the approval of each institutional review board. This study was registered with the University Hospital Medical Information Network database (UMIN000041483). The requirement for written informed consent was waived due to the retrospective design of the study.

### Patients

The details of this study’s eligibility have been previously reported [10]. In summary, the eligibility criteria were as follows: (i) patients older than 20 years, (ii) newly diagnosed or postoperative recurrent NSCLC that was cytologically or pathologically confirmed, and (iii) who were treated with definitive CCRT (≥ 54 Gy in equivalent dose in 2-Gy fractions [EQD2], which was according to one of the inclusion criteria for the PACIFIC trial [3]) between 1 July 2018 and 31 July 2019. Patients with clinical stage I, II and IV NSCLC, according to the 8th edition of the UICC, were excluded from this secondary analysis. Then, we divided the eligible patients into two cohorts: the CCRT+D cohort where at least one course of consolidation durvalumab was administered, and the CCRT cohort without consolidation durvalumab.

### Data collection and endpoints

The data cutoff date was August 31, 2021. Data on survival, patterns of initial recurrence (locoregional recurrence [LR] or distant metastasis [DM]), the relationship of LR to the dose distribution, and durvalumab administration status, were retrospectively collected from medical records. The patient characteristics, tumor characteristics and radiotherapy treatment details that had been collected in the previous reports, were used for analysis: age, sex, Eastern Cooperative Oncology Group performance status (ECOG-PS), smoking history, histology, epidermal growth factor receptor (EGFR) mutation status, anaplastic lymphoma kinase (ALK) rearrangement, c-ros oncogene 1 (ROS1) mutation status, PD-L1 expression (≥ 1% or < 1%) determined by immunohistochemistry using Dako22C3 assay, T category, N category, stage according to Union for International Cancer Control 8^th^ edition, regimen for CCRT, the period from the day of the last radiotherapy to the initiation of durvalumab, irradiation technique (three-dimensional conformal radiotherapy [3D-CRT] or intensity-modulated radiotherapy [IMRT], including the combination of 3D-CRT and IMRT), treatment volume (elective nodal irradiation [ENI] or involved-field irradiation [IFI]), gross tumor volume (GTV; the summed volume of primary tumor and lymph node metastasis), and hospital volume. ENI was defined as the irradiation to the uninvolved lymph-node station. IFI was defined as irradiation without ENI. The present study did not collect data on which IFI was based on involved node station or on geometric expansion of involved node. Hospitals were dichotomized into high volume and low volume according to the median annual number of patients with NSCLC who were treated with definitive CCRT.

Disease progression was defined as any LR or DM diagnosed based on radiological and/or pathological findings. LR was defined as any recurrent tumor in the primary tumor or the hilar, mediastinal, subcarinal, or supraclavicular lymph nodes. Furthermore, LR was classified as in-field recurrence (IF: LR occurring where ≥ 90% of the prescribed dose was irradiated), elective field recurrence (EF: LR occurring where 66–90% of the prescribed dose was irradiated without evidence of IF) and out-of-field recurrence (OF: LR occurring solely where < 66% of the prescribed dose was irradiated without IF or EF). The cut-off values were set assuming 60 Gy as 100% of the prescribed dose, 54 Gy as 90% and 40 Gy as 66%. The central review of CT images at recurrence was not performed. As radiation-induced lung fibrosis occurs gradually 6–12 months after CCRT, whether the loco-regional recurrence located in 90% or 66–90% of the prescribed dose was determined by treating physicians considering radiological changes during the follow-up. DM was classified into brain, bone, lung, liver, adrenal gland, lymph node, pleural or pericardial dissemination/carcinomatous pleurisy, pericarditis and other DM. When LR and DM occurred within 4 weeks, we classified them as simultaneous recurrences of LR and DM (LR + DM). The follow-up period was calculated on the last day of RT. Progression-free survival (PFS) was defined as the period between the last day of radiotherapy and the day of initial disease progression or death from any cause and was censored on the last day of follow-up. OS was defined as the period between the last day of radiotherapy and the day of death from any cause, and was censored on the last day of follow-up.

### Statistical analysis

When comparing the two groups of patient backgrounds, Fisher’s exact test and the Mann–Whitney *U* test were performed to compare categorical and continuous variables, respectively. A univariate logistic regression model was used to estimate the association between each covariate and recurrence pattern, followed by multivariate logistic regression with the stepwise variable selection according to the Akaike information criterion. PFS and OS were calculated using the Kaplan–Meier method. *P* values for the differences between the curves were calculated using the log-rank test. Cox proportional hazard models were used to estimate the effect of each covariate on PFS, followed by a multivariate stepwise Cox regression analysis. Propensity score matching was performed to reduce the effects of selection bias between the CCRT+D and CCRT cohorts. The propensity scores were estimated using logistic regression considering eight covariates: age, sex, ECOG-PS, histology, EGFR mutation status, PD-L1 expression status, stage and GTV volume. The matching procedure was performed using the 2:1 nearest-neighbor matching method with a caliper of 0.20. All *P* values were two-sided, and statistical significance was set at *P* < 0.05. Statistical analyses were performed using R software (version 4.0.2) and the MatchIt package (version 4.3.0).

## RESULTS

### Patient characteristics

One hundred seventy-eight patients were analyzed, including 136 in the CCRT+D cohort and 42 in the CCRT cohort. A flow chart for the patient selection and the reasons for durvalumab omission is shown in Supplemental Fig. 1. There were no significant differences in patient characteristics between the CCRT+D and CCRT cohorts, except for the incidence of ALK rearrangement (Table 1). Most patients (*n* = 166, 93.3%) received 60 Gy in 30 fractions, five patients (2.8%) received more than 60 Gy (range, 65–72 Gy in EQD2) and seven patients (3.9%) received less than 60 Gy (range, 54–57.5 Gy in EQD2).

**Table 1:** Patient characteristics

		**Overall**	**CCRT + D cohort**	**CCRT cohort**	***P* value**
		*n* = 178	*n* = 136	*n* = 42	
Age (years)	median [IQR]	70 [65–75]	70 [64–75]	71 [68–76]	0.42
Sex	Male/Female	144/34	111/25	33/9	0.83
ECOG-PS	0/1/2	112/57/9	87/43/6	25/14/3	0.74
History of smoking	Yes/No	154/24	117/19	37/5	0.93
Histology	Adeno/SqCC/Others	81/70/27	62/55/19	19/15/8	0.70
EGFR mutation	Negative/Positive/Unknown	90/21/67	69/17/50	21/4/17	0.84
PD-L1 expression status	≥ 1%/ < 1%/Unknown	96/39/43	76/30/30	20/9/13	0.48
ALK rearrangement	Negative/Positive/Unknown	97/4/77	80/1/55	17/3/22	0.012
ROS1 mutation	Negative/Positive/Unknown	81/2/95	63/1/72	18/1/23	0.65
T category	T0, X/T1, 2/T3, 4	30/66/82	22/50/64	8/16/18	0.86
N category	N0/1/2/3	11/15/96/56	7/12/72/45	4/3/24/11	0.64
Stage	IIIA/IIIB/IIIC	81/82/15	57/68/11	24/14/4	0.16
Platinum agent	CBDCA/CDDP	148/30	110/26	38/4	0.22
Irradiation technique	3D-CRT/IMRT	133/45	100/36	33/9	0.65
Treatment volume	IFI/ENI	61/117	41/95	20/22	0.058
GTV volume (cm^3^)	median [IQR]	57.1 [29.4–123.4]	55.8 [27.6–127.0]	59.9 [31.2–120.8]	0.75
Hospital volume	High /Low	132/46	103/33	29/13	0.51

*Abbreviations:* Adeno, adenocarcinoma; ALK, anaplastic lymphoma kinase; CBDCA, carboplatin; CCRT, concurrent chemoradiotherapy; CDDP, cisplatin; D, consolidation durvalumab; ECOG-PS, Eastern Cooperative Oncology Group performance status; EGFR, epidermal growth factor receptor; ENI, elective nodal irradiation; GTV, gross tumor volume; IFI, involved field irradiation; IMRT, intensity-modulated radiotherapy; IQR, interquartile range; PD-L1, programmed death-ligand 1; ROS1, c-ros oncogene 1; RT, radiotherapy; SqCC, squamous cell carcinoma; 3D-CRT, three-dimensional conformal radiotherapy.

The median follow-up periods were 25.4 months (interquartile range [IQR], 19.2–30.8 months) in the CCRT+D cohort and 22.9 months (IQR, 12.0–28.8 months) in the CCRT cohort. In the CCRT+D cohort, the median number of courses of durvalumab was 15 (IQR, 5–24). Sixty patients (44.1%) received durvalumab treatment for 12 months. The reasons for durvalumab discontinuation were disease progression in 41 patients (30.1%) and adverse events in 30 patients (22.1%). Consolidation chemotherapy was administered to 10 patients (23.8%) in the CCRT cohort.

### Patterns of initial recurrence and risk factors for LR or DM

Seventy-eight patients (57.4%) in the CCRT+D cohort and 32 patients (76.2%) in the CCRT cohort experienced recurrence during the follow-up period. LR, LR + DM and DM were observed in 28 (20.6%), 12 (8.8%) and 38 (27.9%) patients in the CCRT+D cohort; and in 11 (26.2%), 7 (16.7%) and 14 (33.3%) patients in the CCRT cohort, respectively. Among patients with LR, IF was the most common, followed by OF and EF in both cohorts.

**Fig. 1:**
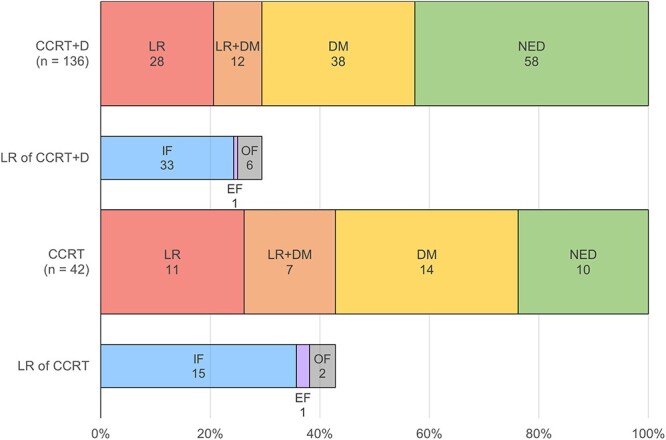
The patterns of initial recurrence in the chemoradiotherapy and consolidation durvalumab (CCRT+D) cohort and the CCRT cohort. *Abbreviations:* LR, locoregional recurrence; DM, distant metastasis; NED, no evidence of disease; IF, in-field recurrence; EF, elective field recurrence; OF, out-of-field recurrence.

The common sites of DM were the brain (16 patients, 11.8%), lungs (12 patients, 8.8%), adrenal glands (10 patients, 7.4%), bone (seven patients, 5.1%), and liver (six patients, 4.4%) in the CCRT+D cohort, and the brain (five patients, 11.9%), liver (five patients, 11.9%), lymph nodes (four patients, 9.5%), lungs (three patients, 7.1%) and bone (three patients, 7.1%) in the CCRT cohort. The details of the recurrence patterns are shown in Figs 1a and b.

Univariate analysis showed that squamous cell carcinoma (odds ratio [OR], 2.42; 95% confidence interval [CI], 1.22–4.90; *P* = 0.027) was significantly associated with an increased risk of LR as the initial recurrence pattern (Supplemental Table 1). Multivariate analysis showed that squamous cell carcinoma (OR, 3.97; 95% CI, 1.82–9.11; *P* = 0.027) and PD-L1 expression < 1% (OR, 2.67; 95% CI, 1.17–6.24; *P* = 0.040) were significantly associated with an increased risk of LR. A GTV ≥ 57 cm^3^ (OR, 0.48; 95% CI, 0.23–0.98; *P* = 0.057) and consolidation durvalumab (OR, 0.47; 95% CI, 0.22–1.02; *P* = 0.051) were marginally associated with a decreased risk of LR.

As for DM, the univariate analysis showed that female sex (OR, 3.67; 95% CI, 1.54–9.11; *P* < 0.001) and EGFR mutation (OR, 4.45; 95% CI, 1.43–15.6; *P* = 0.039) were significantly associated with an increased risk of DM (Supplemental Table 2). Multivariate analysis showed that female sex (OR, 3.67; 95% CI, 1.54–9.11; *P* < 0.001) and EGFR mutation (OR, 4.45; 95% CI, 1.43–15.6; *P* = 0.039) were significantly associated with an increased risk of DM. The association between consolidation durvalumab treatment and the risk of DM was not statistically significant (OR, 0.55; 95% CI, 0.26–1.15; *P* = 0.11).

### Progression-free and overall survival

**Fig. 2:**
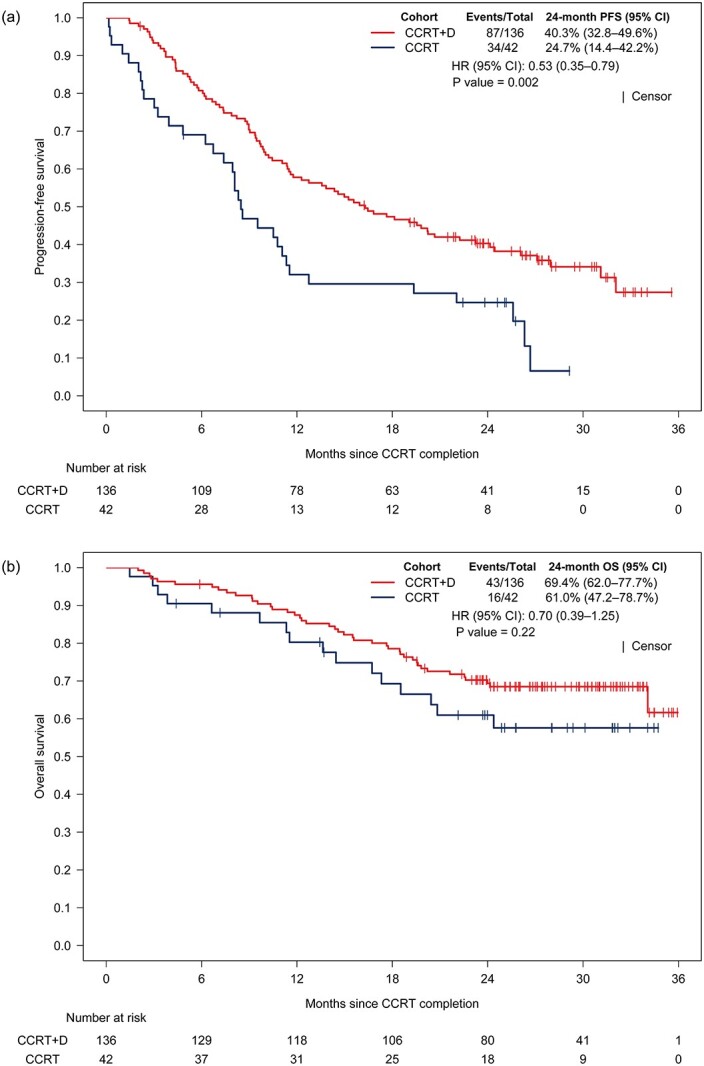
Kaplan–Meier curve of (a) PFS and (b) OS in chemoradiotherapy (CCRT) with durvalumab cohort versus CCRT cohort.

Forty-three patients in the CCRT+D cohort died, and the 24 month PFS and OS rates were 40.3% (95% CI, 32.8–49.6%) and 69.4% (95% CI, 62.0–77.7%), respectively. In the CCRT cohort, 16 patients died and the 24 month PFS and OS rates were 24.7% (95% CI, 14.4–42.2%) and 61% (95% CI, 47.2–78.7%), respectively. There was a significant difference in PFS between the CCRT+D and CCRT cohorts (hazard ratio [HR], 0.53; 95% CI, 0.35–0.79; *P* = 0.002; Fig. 2a). There was no significant difference in OS between the CCRT+D and CCRT cohorts (HR, 0.70; 95% CI, 0.39–1.25; *P* = 0.22; Fig. 2b).

Univariate analysis showed that an ECOG-PS of 2, GTV ≥ 57 cm^3^, and no use of consolidation durvalumab were significantly associated with shorter PFS (Table 2). Multivariate analysis showed that an ECOG-PS of 2 (HR, 2.10; 95% CI, 1.05–4.40; *P* = 0.035) and the use of consolidation durvalumab (HR, 0.52; 95% CI, 0.35–0.78; *P* = 0.003) were significantly associated with PFS.

### Subgroup analysis for the patients with high risk for LR or with high risk for DM

**Fig. 3:**
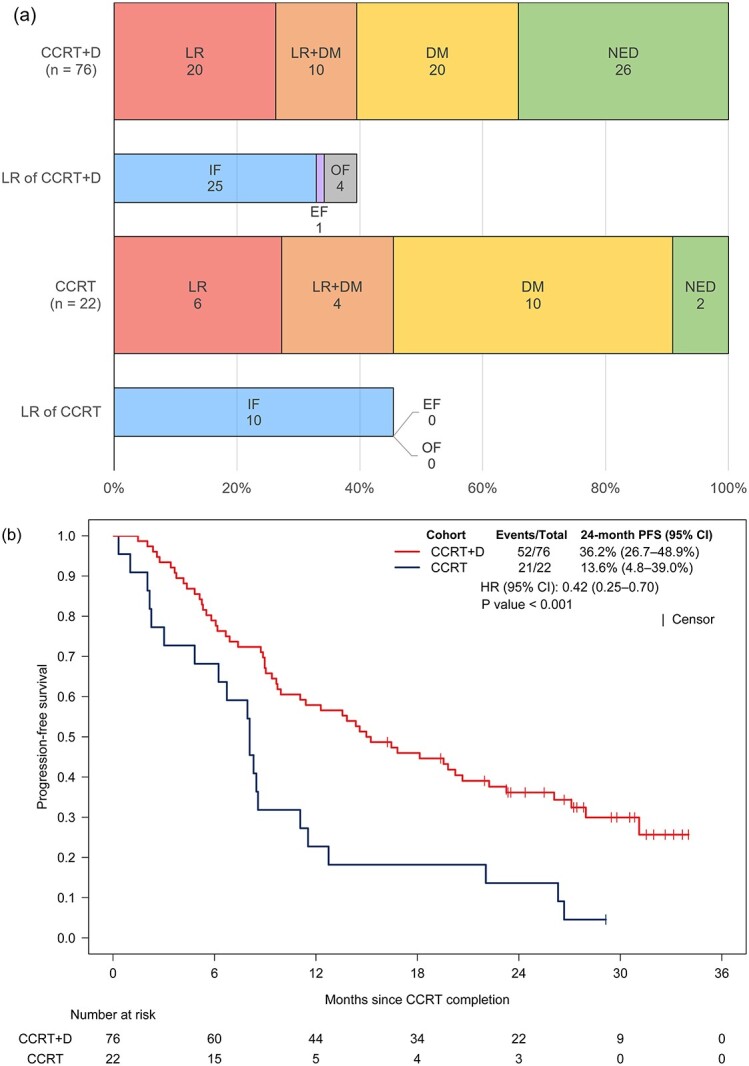

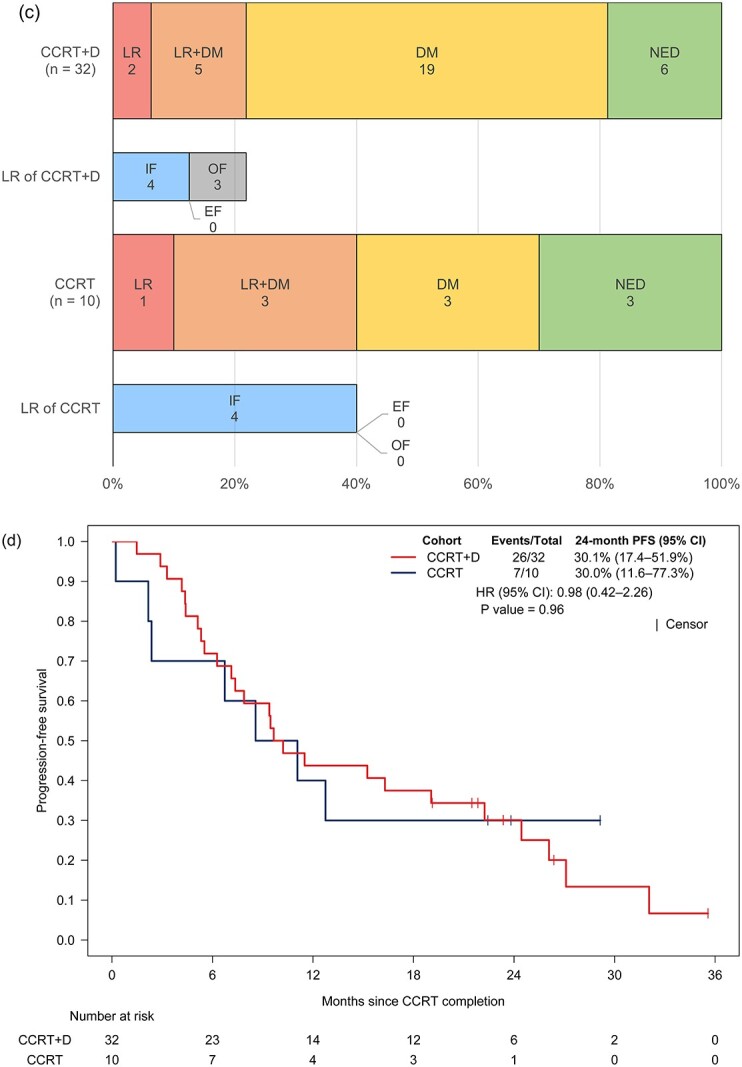
(a) Patterns of initial recurrence and (b) PFS in patients at high risk of local recurrence (squamous cell carcinoma or PD-L1 expression status < 1%), and (c and d) those at high risk of distant metastasis (female or EGFR mutation). The abbreviations are the same as shown in Fig. 2.

Patients with squamous cell carcinoma or PD-L1 expression < 1%, who were identified as having a high risk for LR, presented a lower percentage of DM as initial recurrence in the CCRT+D cohort than in the CCRT cohort (39.5% vs 63.6%), although the percentage of LR was similar in the CCRT+D and CCRT cohorts (39.5% vs 45.5%) (Fig. 3a). Consolidation durvalumab was significantly associated with improved PFS in patients at high risk of LR (HR, 0.42; 95% CI, 0.25–0.70; *P* < 0.001; Fig. 3b). There were no significant differences in patient characteristics between the CCRT+D and CCRT cohorts (Supplemental Table 3).

Patients with EGFR-mutant NSCLC or females, who were identified as high-risk for DM, presented no decrease in the percentage of DM as initial recurrence in CCRT+D compared with the CCRT cohort (75% vs 60%). However, the percentage of LR was lower in the CCRT+D cohort than in the CCRT cohort (21.9% vs 40%) (Fig. 3c). No significant difference in PFS between the CCRT+D and CCRT cohorts was observed in patients at a high risk of DM (HR, 0.98; 95% CI, 0.42–2.26; *P* = 0.96; Fig. 3d). There was no significant difference in patient characteristics between the CCRT+D and CCRT cohorts, except for treatment volume (Supplemental Table 4).

**Table 2:** Univariate and multivariate analysis for PFS

			**Median PFS**	**Univariate analysis**		**Multivariate analysis**	
**Characteristic**		**N**	**(months)**	**HR [95% CI]**	***P* value**	**HR [95% CI]**	***P* value**
Age	< 70 years	77	11.8	*Reference*	0.95		
	≥ 70 years	101	13.8	0.99 [0.69–1.42]			
Sex	Male	144	15.0	*Reference*	0.10		
	Female	34	9.8	1.45 [0.94–2.22]			
ECOG-PS	0	112	15.4	*Reference*	0.035	*Reference*	0.035
	1	57	9.9	1.42 [0.97–2.08]		1.32 [0.89–1.95]	
	2	9	5.3	2.43 [1.17–5.05]		2.10 [1.01–4.40]	
History of smoking	No	24	13.2	*Reference*	0.93		
	Yes	154	13.8	1.02 [0.60–1.73]			
Histology	Adeno	81	16.3	*Reference*	0.19		
	SqCC	70	10.5	1.43 [0.97–2.11]			
	Others	27	11.3	1.19 [0.69–2.05]			
EGFR mutation	Negative	90	13.8	*Reference*	0.20		
	Positive	21	11.5	1.54 [0.89–2.66]			
	Unknown	67	15.0	1.32 [0.90–1.94]			
PD-L1 expression status	≥ 1%	96	13.8	*Reference*	0.96		
	< 1%	39	13.6	1.06 [0.68–1.66]			
	Unknown	43	12.7	1.05 [0.67–1.62]			
Stage	IIIA	81	12.8	*Reference*	0.093		
	IIIB	82	14.8	1.10 [0.75–1.60]			
	IIIC	15	8.3	2.08 [1.13–3.85]			
Irradiation technique	3D-CRT	133	13.8	*Reference*	0.64		
	IMRT	45	13.6	1.11 [0.73–1.66]			
Treatment volume	IFI	61	13.6	*Reference*	0.68		
	ENI	117	13.8	1.08 [0.74–1.59]			
GTV volume	< 57 cm^3^	89	16.5	*Reference*	0.020	*Reference*	0.072
	≥ 57 cm^3^	89	9.5	1.53 [1.07–2.19]		1.44 [1.00–2.09]	
Hospital volume	High	132	15.2	*Reference*	0.15		
	Low	46	11.3	1.35 [0.91–2.00]			
Consolidation durvalumab	No	42	8.5	*Reference*	0.003	*Reference*	0.003
	Yes	136	16.3	0.53 [0.35–0.79]		0.52 [0.35–0.78]	

*Abbreviations:* CI, confidence interval; HR, hazard ratio; MST, median survival. The other abbreviations are the same as those in Table 1.

### Propensity score-matched analysis for CCRT+D cohort vs CCRT cohort

After propensity score matching, the patient backgrounds, including eight covariates, were well balanced between the matched CCRT+D cohort (*n* = 72) and the matched CCRT cohort (*n* = 40) (Supplemental Table 5, Supplemental Fig. 2).

**Fig. 4:**
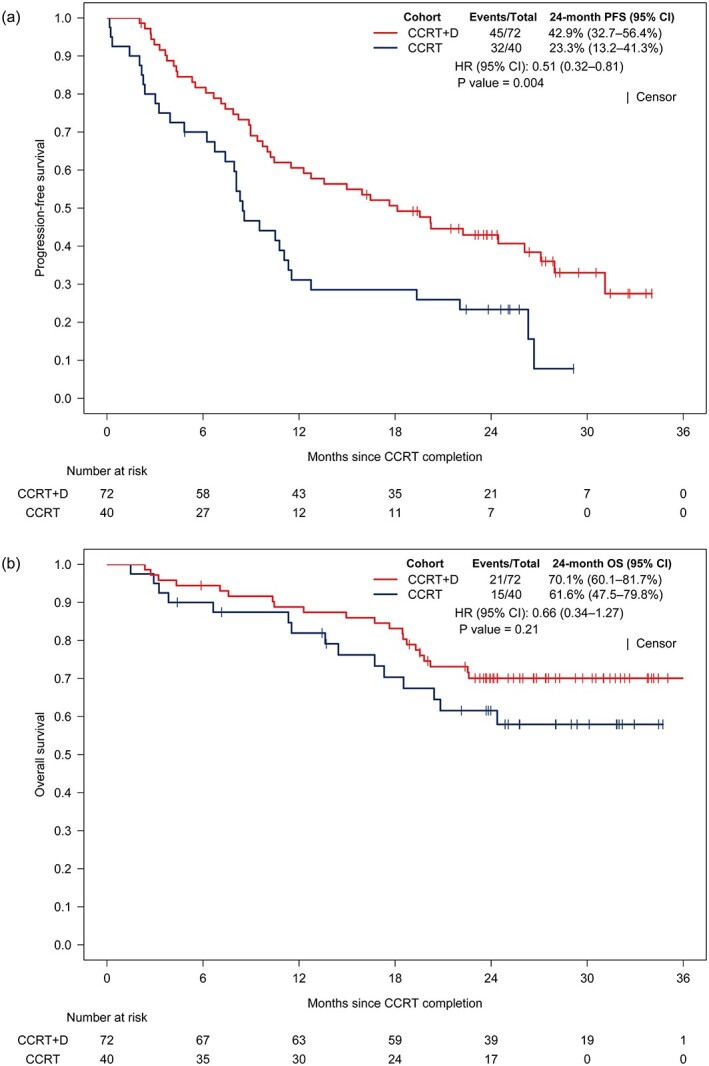
Kaplan–Meier curves of (a) PFS and (b) OS in CCRT with consolidation durvalumab cohort and CCRT cohort after propensity-score matched analysis.

The 24 month PFS rate was 42.9% (95% CI, 32.7–56.4%), and the 24 month OS rate was 70.1% (95% CI, 60.1–81.7%) in the CCRT+D cohort. The 24 month PFS rate was 23.3% (95% CI, 13.2–41.3%) and the 24 month OS rate was 61.6% (95% CI, 47.5–79.8%) in the CCRT cohort. The CCRT+D cohort showed significantly improved PFS compared to the CCRT cohort (HR, 0.51; 95% CI, 0.32–0.81; *P* = 0.004; Fig. 4a), and no significant difference in OS (HR, 0.66; 95% CI, 0.34–1.27; *P* = 0.21; Fig. 4b).

## DISCUSSION

As more than 4 years have passed since the PACIFIC trial was first reported, increasing attention has been paid to the real-world outcomes of CCRT with consolidation durvalumab among patients with locally advanced NSCLC. This multi-institutional retrospective study presented the detailed patterns of recurrence in Japanese patients with newly diagnosed or recurrent stage III NSCLC who received CCRT with or without consolidation durvalumab. Our study showed that DM was the most common recurrence pattern, and consolidation durvalumab significantly improved PFS in this population. Two strengths distinguish our study from the PACIFIC trial and previous reports. First, we collected information on the relationship between consolidation durvalumab and recurrence patterns with the irradiated dose of the LR site. Second, we collected data on patients who received CCRT with and without consolidation durvalumab in the same period, and matched the CCRT with consolidation durvalumab cohort with the CCRT without consolidation durvalumab cohort using propensity score-matched analysis.

In our study, we found that squamous cell carcinoma and PD-L1 expression of < 1% were associated with LR. As shown in a previous report, failure patterns differ in histology, and squamous cell carcinoma has a higher risk of LR than adenocarcinoma [11]. In the post-hoc analysis of the PACIFIC trial, OS and PFS benefits were observed in the durvalumab group across all PD-L1 subgroups, except for OS in patients with PD-L1 expression < 1% [4, 12]; however, few reports have focused on the association between recurrence patterns and PD-L1 expression status. Shaverdian *et al.* reported that histology and PD-L1 expression ≥ 1% were not associated with locoregional control in 66 patients with stage III NSCLC who received CCRT and consolidation durvalumab [8]. In contrast, our study showed that CCRT with consolidation durvalumab decreased DM as an initial recurrence, and was significantly associated with improved PFS among patients with squamous cell carcinoma or PD-L1 expression < 1% compared with CCRT without durvalumab, whereas LR, especially IF, remained high. Yoneda *et al.* reported that tumoral PD-L1 expression levels were upregulated after CCRT, and that there was no significant correlation between baseline and post-CCRT PD-L1 expression [13]. Therefore, future research that considers this alteration would clarify the significance of PD-L1 expression as a biomarker.

The different proportions of IFI and ENI between CCRT+D group and CCRT group could affect the proportions of EF and OF. Therefore, a direct comparison of EF and OF between these two groups could not be made. However, IF, which was the most common pattern of LR, was not affected by how much proportion of the patients received IFI or ENI. In both CCRT+D and CCRT groups, the issue was LR occurring where ≥ 90% of the prescribed dose was irradiated. Further improvement of loco-regional control is still a major problem even after the introduction of consolidation durvalumab. RTOG0617, a randomized phase 3 trial, demonstrated that dose-escalated radiotherapy showed decreased OS and increased toxicity compared to standard-dose radiotherapy in stage III NSCLC [14]. According to RTOG0617, the simple application of dose-escalated radiotherapy to any patient was found to be of limited value [15, 16]. Selected patients who are at high risk for LR in our study may be good candidates for the dose escalation using advanced radiotherapy techniques. Simultaneous integrated boost IMRT technique or stereotactic body radiotherapy boost technique, which increases the dose to GTV or residual lesions, would contribute to improving locoregional control [17, 18]. The routinely adaptive radiotherapy strategy could reduce the dose to surrounding normal tissues [19].

It is well known that EGFR mutations in NSCLC frequently occur in East Asians, females, non-smokers and adenocarcinomas [20, 21]. Non-squamous cell carcinomas that are positive for EGFR mutations are susceptible to developing DM [22–24]. Our results regarding the prognostic factors for DM are consistent with those of previous reports. As for OS in the PACIFIC trial, the HRs of the population with EGFR mutation or ALK rearrangement positive, negative and unknown, were 0.85 (95% CI, 0.37–1.97), 0.66 (95% CI, 0.52–0.84), and 0.85 (95% CI, 0.57–1.24), respectively [4]. These results suggest that the survival benefit of CCRT with consolidation durvalumab might be reduced among patients with EGFR-mutant NSCLC. However, this was unclear in the PACIFIC trial because the number of patients with EGFR-mutant NSCLC was small, and because of the imbalance of the patient background among the subgroups. Hellyer *et al.* reported 36 patients with stage III NSCLC treated with consolidation durvalumab [25]. Patients with tumor mutations in the erythroblastic leukemia viral oncogene homolog (ErbB) family, including EGFR and ErbB2, had a significantly shorter disease-free survival than those with wild-type EGFR or ErbB2. Our study indicated a tendency equivalent to that in the PACIFIC trial and the study reported by Hellyer *et al.*; all findings supported the view that consolidation durvalumab should not be administered to patients with stage III EGFR mutation-positive NSCLC, which was recommended by Aredo *et al.* [26] In our study, the incidence of DM remained high, even after the consolidation of durvalumab. Since the ADAURA trial showed that adjuvant osimertinib after resection significantly improved PFS [27], the combination of local therapy and targeted therapy could be efficacious for stage III NSCLC harboring mutations in EGFR. The ongoing LAURA trial, which is investigating osimertinib following CCRT [28], may clarify the optimal treatment strategy for this population.

In this study we compared two cohorts (CCRT+D cohort and CCRT cohort) during the same time period. This allowed us to avoid historical control bias [9]. In addition, propensity score matching reduced the variation in pretreatment factors between the two cohorts. However, our study is inherently biased in treatment selection: disease progression and adverse events after radiotherapy affect the decision to use durvalumab and are major determinants of PFS. Other factors, including tumor histology, recurrent tumor and synchronous cancer, might also affect the decision and outcomes. Therefore, inevitably, the PFS of the CCRT cohort would be worse than that of the CCRT+D cohort, thereby potentially overestimating the effect of durvalumab. Nonetheless, it is noteworthy that the difference in OS between the two cohorts in this study was not significant, although a longer follow-up is needed to obtain mature OS results.

Our study had other limitations that should be acknowledged. First, EGFR mutational status and PD-L1 expression status were unavailable in 38% and 24% of the patients, respectively. Second, PFS was scheduled to start on the last day of radiotherapy, whereas the PACIFIC trial set it as the day of randomization, which was 1 to 42 days from the last day of radiotherapy. Third, GTV ≥ 57 cm^3^ was marginally associated with an increased risk of disease progression, while it was marginally associated with a decreased risk of LR. There were two possible reasons for this: (i) the collected patterns of recurrence were limited to the initial recurrence, and (ii) the GTV volume included both the primary tumor and the nodal volumes. As for the former reason, our analysis could not consider either LR occurring after DM or DM after LR. The prognostic value of the GTV volume for LR could have been underestimated due to the earlier occurrence of DM. For the latter reason, it has been reported that primary tumors are more likely to fail than lymph nodes [11]. As we could not differentiate cases with large primary tumors plus small lymph nodes from those with small primary tumors plus large lymph node metastasis, the underlying bias in the volumes of primary tumors and lymph nodes might have affected the results. Furthermore, the actuarial irradiated doses for GTV such as dose minimum were unavailable. We need further investigation to evaluate the association between the actuarial irradiated dose to target volume and the detailed recurrence patterns.

## CONCLUSION

In conclusion, this multi-institutional study of Japanese patients with stage III NSCLC treated with CCRT showed that consolidation durvalumab decreased both LR and DM and significantly improved PFS. However, IF is still a major problem after CCRT and consolidation of durvalumab, as well as DM.

## AUTHOR STATEMENT

Noriko Kishi: data curation, formal analysis, investigation, software, visualization and writing (original draft); Yukinori Matsuo: funding acquisition, project administration, methodology, software and writing (review and editing); Takashi Shintani: conceptualization, investigation, validation and writing (review and editing); Masakazu Ogura: resources and writing (review and editing); Takamasa Mitsuyoshi: resources and writing (review and editing); Norio Araki: resources and writing (review and editing); Kota Fujii: resources and writing (review and editing); Setsuko Okumura: resources and writing (review and editing); Kiyoshi Nakamatsu: resources and writing (review and editing); Takahiro Kishi: resources and writing (review and editing); Tomoko Atsuta: resources and writing (review and editing); Takashi Sakamoto: resources and writing (review and editing); Shuji Ohtsu: resources and writing (review and editing); Tomohiro Katagiri: resources and writing (review and editing); Masaru Narabayashi: resources and writing (review and editing); Satsuki Fujishiro: resources and writing (review and editing); Yusuke Iizuka: resources and writing (review and editing); Hiroaki Ozasa: writing (review and editing); Toyohiro Hirai: writing (review and editing); Takashi Mizowaki: supervision and writing (review and editing).

## Supplementary Material

KROSGdurvalumab_Supplementarymaterials_20220525_rrac057

## References

[1] Aupérin A, Le Péchoux C, Rolland E et al. Meta-analysis of concomitant versus sequential radiochemotherapy in locally advanced non-small-cell lung cancer. J Clin Oncol 2010;28:2181–90. 20351327 10.1200/JCO.2009.26.2543

[2] National Comprehensive Cancer Network . Non-Small Cell Lung Cancer (Version 1.2022). https://www.nccn.org/professionals/physician_gls/pdf/nscl.pdf Accessed February 22 2022.

[3] Antonia SJ, Villegas A, Daniel D et al. Overall survival with Durvalumab after Chemoradiotherapy in stage III NSCLC. N Engl J Med 2018;379:2342–50. 30280658 10.1056/NEJMoa1809697

[4] Spigel DR, Faivre-Finn C, Gray JE et al. Five-year survival outcomes from the PACIFIC trial: Durvalumab after Chemoradiotherapy in stage III non-small-cell lung cancer. J Clin Oncol 2022;40:1301–11. 35108059 10.1200/JCO.21.01308 PMC9015199

[5] Taugner J, Eze C, Käsmann L et al. Pattern-of-failure and salvage treatment analysis after chemoradiotherapy for inoperable stage III non-small cell lung cancer. Radiat Oncol 2020;15:148 Published 2020 Jun 9. 32517716 10.1186/s13014-020-01590-8 PMC7285541

[6] Abe T, Saito S, Iino M et al. Effect of durvalumab on local control after concurrent chemoradiotherapy for locally advanced non-small cell lung cancer in comparison with chemoradiotherapy alone. Thorac Cancer 2021;12:245–50. 33289347 10.1111/1759-7714.13764 PMC7812072

[7] Sankar K, Bryant AK, Strohbehn GW et al. Real world outcomes versus clinical trial results of Durvalumab maintenance in veterans with stage III non-small cell lung cancer. Cancers (Basel) 2022;14:614. 35158881 10.3390/cancers14030614 PMC8833364

[8] Shaverdian N, Offin M, Shepherd AF et al. The impact of Durvalumab on local-regional control in stage III NSCLCs treated with Chemoradiation and on KEAP1-NFE2L2-mutant Tumors. J Thorac Oncol 2021;16:1392–402. 33992811 10.1016/j.jtho.2021.04.019 PMC8316395

[9] Hansen RN, Zhang Y, Seal B et al. Long-term survival trends in patients with unresectable stage III non-small cell lung cancer receiving chemotherapy and radiation therapy: a SEER cancer registry analysis. BMC Cancer 2020;20:276. 32248816 10.1186/s12885-020-06734-3 PMC7132866

[10] Shintani T, Kishi N, Matsuo Y et al. Incidence and risk factors of symptomatic radiation pneumonitis in non-small-cell lung cancer patients treated with concurrent chemoradiotherapy and consolidation Durvalumab. Clin Lung Cancer 2021;22:401–10. 33678582 10.1016/j.cllc.2021.01.017

[11] Nygård L, Vogelius IR, Fischer BM et al. A competing risk model of first failure site after definitive Chemoradiation therapy for locally advanced non-small cell lung cancer. J Thorac Oncol 2018;13:559–67. 29355615 10.1016/j.jtho.2017.12.011

[12] Paz-Ares L, Spira A, Raben D et al. Outcomes with durvalumab by tumour PD-L1 expression in unresectable, stage III non-small-cell lung cancer in the PACIFIC trial. Ann Oncol 2020;31:798–806. 32209338 10.1016/j.annonc.2020.03.287 PMC8412232

[13] Yoneda K, Kuwata T, Kanayama M et al. Alteration in tumoural PD-L1 expression and stromal CD8-positive tumour-infiltrating lymphocytes after concurrent chemo-radiotherapy for non-small cell lung cancer. Br J Cancer 2019;121:490–6. 31388183 10.1038/s41416-019-0541-3 PMC6738061

[14] Bradley JD, Hu C, Komaki RR et al. Long-term results of NRG oncology RTOG 0617: standard- versus high-dose Chemoradiotherapy with or without Cetuximab for Unresectable stage III non-small-cell lung cancer. J Clin Oncol 2020;38:706–14. 31841363 10.1200/JCO.19.01162 PMC7048161

[15] Eaton BR, Pugh SL, Bradley JD et al. Institutional Enrollment and survival among NSCLC patients receiving Chemoradiation: NRG oncology radiation therapy oncology group (RTOG) 0617. J Natl Cancer Inst 2016;108:djw034. 27206636 10.1093/jnci/djw034 PMC6059090

[16] Favre-Finn C . Dose escalation in lung cancer: have we gone full circle? Lancet Oncol 2015;16:125–7. 25601340 10.1016/S1470-2045(15)70001-X

[17] Zhang Q, Cai XW, Feng W et al. Dose-escalation by hypofractionated simultaneous integrated boost IMRT in unresectable stage III non-small-cell lung cancer. BMC Cancer 2022;22:96. 35065627 10.1186/s12885-021-09099-3 PMC8783483

[18] Higgins KA, Pillai RN, Chen Z et al. Concomitant chemotherapy and radiotherapy with SBRT boost for Unresectable stage III non-small cell lung cancer: a phase I study. J Thorac Oncol 2017;12:1687–95. 28919394 10.1016/j.jtho.2017.07.036

[19] Meng Y, Luo W, Xu H et al. Adaptive intensity-modulated radiotherapy with simultaneous integrated boost for stage III non-small cell lung cancer: is a routine adaptation beneficial? Radiother Oncol 2021;158:118–24. 33636232 10.1016/j.radonc.2021.02.019

[20] Kosaka T, Yatabe Y, Endoh H et al. Mutations of the epidermal growth factor receptor gene in lung cancer: biological and clinical implications. Cancer Res 2004;64:8919–23. 15604253 10.1158/0008-5472.CAN-04-2818

[21] Shigematsu H, Lin L, Takahashi T et al. Clinical and biological features associated with epidermal growth factor receptor gene mutations in lung cancers. J Natl Cancer Inst 2005;97:339–46. 15741570 10.1093/jnci/dji055

[22] Shin DY, Na II, Kim CH et al. EGFR mutation and brain metastasis in pulmonary adenocarcinomas. J Thorac Oncol 2014;9:195–9. 24419416 10.1097/JTO.0000000000000069

[23] Fujimoto D, Ueda H, Shimizu R et al. Features and prognostic impact of distant metastasis in patients with stage IV lung adenocarcinoma harboring EGFR mutations: importance of bone metastasis. Clin Exp Metastasis 2014;31:543–51. 24682604 10.1007/s10585-014-9648-3

[24] Yagishita S, Horinouchi H, Katsui Taniyama T et al. Epidermal growth factor receptor mutation is associated with longer local control after definitive chemoradiotherapy in patients with stage III nonsquamous non-small-cell lung cancer. Int J Radiat Oncol Biol Phys 2015;91:140–8. 25442336 10.1016/j.ijrobp.2014.08.344

[25] Hellyer JA, Aredo JV, Das M et al. Role of consolidation Durvalumab in patients with EGFR- and HER2-mutant Unresectable stage III NSCLC. J Thorac Oncol 2021;16:868–72. 33539970 10.1016/j.jtho.2020.12.020

[26] Aredo JV, Hellyer JA, Neal JW et al. Consolidation Durvalumab should not be administered to patients with stage III EGFR-mutant NSCLC. J Thorac Oncol 2021;16:1994–8. 34809803 10.1016/j.jtho.2021.07.033

[27] Wu YL, Tsuboi M, He J et al. Osimertinib in resected EGFR-mutated non-small-cell lung cancer. N Engl J Med 2020;383:1711–23. 32955177 10.1056/NEJMoa2027071

[28] Lu S, Casarini I, Kato T et al. Osimertinib maintenance after definitive chemoradiation in patients with unresectable EGFR mutation positive stage III non-small-cell lung cancer: LAURA trial in progress. Clin Lung Cancer 2021;22:371–5. 33558193 10.1016/j.cllc.2020.11.004

