# Supplementary material for: Recurrence patterns and progression-free survival after chemoradiotherapy with or without consolidation durvalumab for stage III non-small cell lung cancer
Source: J Radiat Res. 2022 Sep 22;64(1):142–53. doi: 10.1093/jrr/rrac057 (PMC9855316; doi:10.1093/jrr/rrac057)
Supplement: KROSGdurvalumab_Supplementarymaterials_20220525_rrac057 [file krosgdurvalumab_supplementarymaterials_20220525_rrac057.docx]

# Supplementary files

**

**

**Supplemental Figure 1.** Flowchart of patient inclusion and exclusion.

**
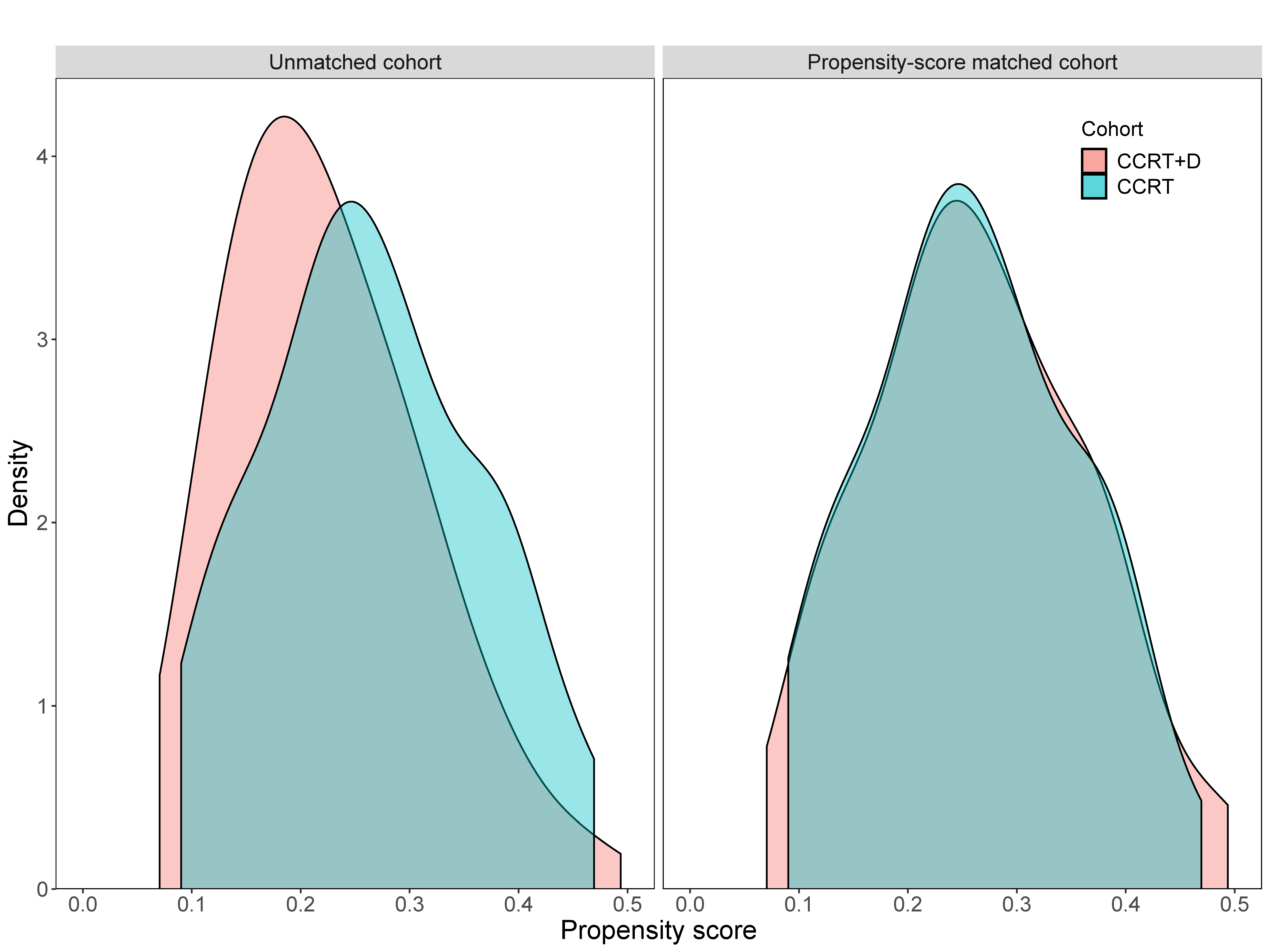
**

**Supplemental Figure 2.** Density plots of propensity scores before and after propensity score matching.

**Supplemental Table 1.** Univariate and multivariate analyses for locoregional recurrence as the initial recurrence.

|  |  | **Univariate analysis** | | | | **Multivariate analysis** | |
| --- | --- | --- | --- | --- | --- | --- | --- |
| **Characteristic** |  | **Yes** | **No** | **OR [95% CI]** | **P value** | **OR [95% CI]** | **P value** |
| Age | < 70 years | 23 | 54 | *Reference* | 0.50 |  |  |
|  | ≥ 70 years | 35 | 66 | 1.25 [0.66–2.37] |  |  |  |
| Sex | Male | 49 | 95 | *Reference* | 0.40 |  |  |
|  | Female | 9 | 25 | 0.70 [0.29–1.56] |  |  |  |
| ECOG-PS | 0 | 36 | 76 | *Reference* | 0.31 |  |  |
|  | 1 | 17 | 40 | 0.90 [0.44–1.78] |  |  |  |
|  | 2 | 5 | 4 | 2.64 [0.66–11.2] |  |  |  |
| History of smoking | No | 5 | 19 | *Reference* | 0.19 |  |  |
|  | Yes | 53 | 101 | 1.99 [0.75–6.28] |  |  |  |
| Histology | Adeno | 20 | 61 | Ref | 0.027 | *Reference* | 0.027 |
|  | SqCC | 31 | 39 | 2.42 [1.22–4.90] |  | 3.97 [1.82–9.11] |  |
|  | Others | 7 | 20 | 1.07 [0.37–2.82] |  | 1.63 [0.51–4.96] |  |
| EGFR mutation | Negative | 31 | 59 | *Reference* | 0.37 |  |  |
|  | Positive | 4 | 17 | 0.45 [0.12–1.34] |  |  |  |
|  | Unknown | 23 | 44 | 0.99 [0.51–1.93] |  |  |  |
| PD-L1 expression status | ≥ 1% | 29 | 67 | *Reference* | 0.11 | *Reference* | 0.040 |
|  | < 1% | 18 | 21 | 1.98 [0.92–4.28] |  | 2.67 [1.17–6.24] |  |
|  | Unknown | 11 | 32 | 0.79 [0.34–1.76] |  | 0.79 [0.31–1.90] |  |
| Stage | IIIA | 25 | 56 | *Reference* | 0.47 |  |  |
|  | IIIB | 26 | 56 | 1.04 [0.54–2.02] |  |  |  |
|  | IIIC | 7 | 8 | 1.96 [0.62–6.06] |  |  |  |
| Irradiation technique | 3D-CRT | 44 | 89 | *Reference* | 0.81 |  |  |
|  | IMRT | 14 | 31 | 0.91 [0.43–1.87] |  |  |  |
| Treatment volume | IFI | 17 | 44 | *Reference* | 0.33 |  |  |
|  | ENI | 41 | 76 | 1.40 [0.72–2.79] |  |  |  |
| GTV volume | < 57 cm^3^ | 32 | 57 | *Reference* | 0.34 | *Reference* | 0.057 |
|  | ≥ 57 cm^3^ | 26 | 63 | 0.74 [0.39–1.38] |  | 0.48 [0.23–0.98] |  |
| Hospital volume | High | 43 | 89 | *Reference* | 1.00 |  |  |
|  | Low | 15 | 31 | 1.00 [0.48–2.03] |  |  |  |
| Consolidation durvalumab | No | 18 | 24 | *Reference* | 0.10 | *Reference* | 0.051 |
|  | Yes | 40 | 96 | 0.56 [0.27–1.14] |  | 0.47 [0.22–1.02] |  |

*Abbreviations:* OR, odds ratio. The abbreviations are the same as those listed in Tables 1 and 2.

**Supplemental Table 2.** Univariate and multivariate analyses for distant metastasis as initial recurrence.

|  |  | **Univariate analysis** | | | | **Multivariate analysis** | |
| --- | --- | --- | --- | --- | --- | --- | --- |
| **Characteristic** |  | **Yes** | **No** | **OR [95% CI]** | **P value** | **OR [95% CI]** | **P value** |
| Age | < 70 years | 33 | 44 | *Reference* | 0.48 |  |  |
|  | ≥ 70 years | 38 | 63 | 0.80 [0.44–1.47] |  |  |  |
| Sex | Male | 47 | 97 | *Reference* | < 0.001 | *Reference* | < 0.001 |
|  | Female | 24 | 10 | 4.95 [2.25–11.6] |  | 3.67 [1.54–9.11] |  |
| ECOG-PS | 0 | 42 | 70 | *Reference* | 0.70 |  |  |
|  | 1 | 25 | 32 | 1.30 [0.68–2.49] |  |  |  |
|  | 2 | 4 | 5 | 1.33 [0.31–5.31] |  |  |  |
| History of smoking | No | 13 | 11 | *Reference* | 0.12 |  |  |
|  | Yes | 58 | 96 | 0.51 [0.21–1.22] |  |  |  |
| Histology | Adeno | 33 | 48 | *Reference* | 0.94 |  |  |
|  | SqCC | 28 | 42 | 0.97 [0.50–1.86] |  |  |  |
|  | Others | 10 | 17 | 0.86 [0.34–2.08] |  |  |  |
| EGFR mutation | Negative | 29 | 61 | *Reference* | 0.001 | *Reference* | 0.039 |
|  | Positive | 16 | 5 | 6.73 [2.38–22.2] |  | 4.45 [1.43–15.6] |  |
|  | Unknown | 26 | 41 | 1.33 [0.69–2.59] |  | 1.44 [0.73–2.88] |  |
| PD-L1 expression status | ≥ 1% | 37 | 59 | *Reference* | 0.66 |  |  |
|  | < 1% | 18 | 21 | 1.36 [0.64–2.90] |  |  |  |
|  | Unknown | 16 | 27 | 0.94 [0.44–1.97] |  |  |  |
| Stage | IIIA | 31 | 50 | *Reference* | 0.25 |  |  |
|  | IIIB | 31 | 51 | 0.98 [0.52–1.85] |  |  |  |
|  | IIIC | 9 | 6 | 2.42 [0.80–7.85] |  |  |  |
| Irradiation technique | 3D-CRT | 53 | 80 | *Reference* | 0.99 |  |  |
|  | IMRT | 18 | 27 | 1.01 [0.50–2.00] |  |  |  |
| Treatment volume | IFI | 23 | 38 | *Reference* | 0.67 |  |  |
|  | ENI | 48 | 69 | 1.15 [0.61–2.19] |  |  |  |
| GTV volume | < 57 cm^3^ | 33 | 56 | *Reference* | 0.44 |  |  |
|  | ≥ 57 cm^3^ | 38 | 51 | 1.26 [0.69–2.31] |  |  |  |
| Hospital volume | High | 50 | 82 | *Reference* | 0.35 |  |  |
|  | Low | 21 | 25 | 1.38 [0.70–2.72] |  |  |  |
| Consolidation durvalumab | No | 21 | 21 | *Reference* | 0.13 | *Reference* | 0.11 |
|  | Yes | 50 | 86 | 0.58 [0.29–1.17] |  | 0.55 [0.26–1.15] |  |

The abbreviations are the same as shown in Tables 1 and 2.

**Supplemental Table 3.** Characteristics of patients at high risk of locoregional recurrence (squamous cell carcinoma or PD-L1 expression status < 1%).

|  |  | **Overall** | **CCRT+D cohort** | **CCRT cohort** | **P value** |
| --- | --- | --- | --- | --- | --- |
| No. of patients |  | 98 | 76 | 22 |  |
| Age | < 70 years/≥ 70 years | 43/55 | 34/42 | 9/13 | 0.94 |
| Sex | Male/Female | 83/15 | 67/9 | 16/6 | 0.15 |
| ECOG-PS | 0/1/2 | 56/35/7 | 46/26/4 | 10/9/3 | 0.28 |
| History of smoking | Yes/No | 91/7 | 71/5 | 20/2 | 1.00 |
| Histology | Adeno/SqCC/Others | 24/70/4 | 19/55/2 | 5/15/2 | 0.40 |
| EGFR mutation | Negative/Positive/Unknown | 46/8/44 | 37/33/6 | 9/11/2 | 0.81 |
| PD-L1 expression status | ≥ 1%/< 1%/Unknown | 44/39/15 | 35/30/11 | 9/9/4 | 0.88 |
| ALK rearrangement | Negative/Positive/Unknown | 52/0/46 | 43/0/33 | 9/0/13 | 0.29 |
| ROS1 mutation | Negative/Positive/Unknown | 38/0/60 | 31/0/45 | 7/0/15 | 0.61 |
| T category | T0, X/T1, 2/T3, 4 | 9/33/56 | 8/23/45 | 1/10/11 | 0.35 |
| N category | N0/1/2/3 | 7/13/52/26 | 4/12/40/20 | 3/1/12/6 | 0.35 |
| Stage | IIIA/IIIB/IIIC | 4/46/8 | 33/37/6 | 11/9/2 | 0.81 |
| Platinum agent | CBDCA/CDDP | 82/16 | 62/14 | 20/2 | 0.48 |
| Irradiation technique | 3D-CRT/IMRT | 72/26 | 56/20 | 16/6 | 1.00 |
| Treatment volume | IFI/ENI | 32/66 | 21/55 | 11/11 | 0.087 |
| GTV volume | < 57 cm^3^/≥ 57 cm^3^ | 40/58 | 32/44 | 8/14 | 0.81 |
| Hospital volume | High/Low | 71/27 | 56/20 | 15/7 | 0.81 |

The abbreviations are the same as in Table 1.

**Supplemental Table 4.** Characteristics of patients at high risk of distant metastasis (female sex or EGFR mutation-positive NSCLC).

|  |  | **Overall** | **CCRT+D cohort** | **CCRT cohort** | **P value** |
| --- | --- | --- | --- | --- | --- |
| No. of patients |  | 42 | 32 | 10 |  |
| Age | < 70 years/≥ 70 years | 19/23 | 15/17 | 4/6 | 0.59 |
| Sex | Male/Female | 8/34 | 25/7 | 1/9 | 0.71 |
| ECOG-PS | 0/1/2 | 30/1/2 | 23/7/2 | 7/3/0 | 0.66 |
| History of smoking | Yes/No | 25/17 | 18/14 | 7/3 | 0.69 |
| Histology | Adeno/SqCC/Others | 27/8/7 | 6/2/2 | 21/6/5 | 0.94 |
| EGFR mutation | Negative/Positive/Unknown | 14/21/7 | 10/17/5 | 4/4/2 | 0.77 |
| PD-L1 expression status | ≥ 1%/< 1%/Unknown | 23/12/7 | 19/7/6 | 4/5/1 | 0.23 |
| ALK rearrangement | Negative/Positive/Unknown | 30/2/10 | 24/1/7 | 6/1/3 | 0.55 |
| ROS1 mutation | Negative/Positive/Unknown | 25/0/17 | 19/0/13 | 6/0/4 | 1.00 |
| T category | T0, X/T1, 2/T3, 4 | 5/21/16 | 4/17/11 | 1/4/5 | 0.67 |
| N category | N0/1/2/3 | 3/1/22/16 | 2/0/16/14 | 1/1/6/2 | 0.20 |
| Stage | IIIA/IIIB/IIIC | 18/20/4 | 12/17/3 | 6/3/1 | 0.42 |
| Platinum agent | CBDCA/CDDP | 32/10 | 24/8 | 8/2 | 1.00 |
| Irradiation technique | 3D-CRT/IMRT | 30/12 | 24/8 | 6/4 | 0.61 |
| Treatment volume | IFI/ENI | 14/28 | 7/25 | 7/3 | 0.015 |
| GTV volume | < 57 cm^3^/≥ 57 cm^3^ | 24/18 | 20/12 | 4/6 | 0.37 |
| Hospital volume | High/Low | 33/9 | 27/5 | 6/4 | 0.23 |

The abbreviations are the same as in Table 1.

**Supplemental Table 5.** Patient characteristics after propensity score matching.

|  |  | **Overall** | **CCRT+D cohort** | **CCRT cohort** | **P value** | **SMD** |
| --- | --- | --- | --- | --- | --- | --- |
| No. of patients |  | 112 | 72 | 40 |  |  |
| Age | < 70 years/≥ 70 years | 45/67 | 30/42 | 15/25 | 0.82 | 0.085 |
| Sex | Male/Female | 88/24 | 57/15 | 31/9 | 1.00 | 0.040 |
| ECOG-PS | 0/1/2 | 74/34/4 | 49/21/2 | 25/13/2 | 0.75 | 0.145 |
| History of smoking | Yes/No | 95/17 | 60/12 | 35/5 | 0.75 | 0.118 |
| Histology | Adeno/SqCC/Others | 52/40/20 | 33/26/13 | 19/14/7 | 0.99 | 0.033 |
| EGFR mutation | Negative/Positive/Unknown | 57/13/42 | 36/9/27 | 21/4/15 | 0.92 | 0.082 |
| PD-L1 expression status | ≥ 1%/< 1%/Unknown | 56/16/30 | 36/17/19 | 20/9/11 | 0.99 | 0.032 |
| ALK rearrangement | Negative/Positive/Unknown | 56/4/52 | 39/1/32 | 17/3/20 | 0.17 | 0.350 |
| ROS1 mutation | Negative/Positive/Unknown | 50/2/60 | 32/1/39 | 18/1/21 | 0.91 | 0.083 |
| T category | T0, X/T1, 2/T3, 4 | 20/43/49 | 12/29/31 | 8/14/18 | 0.83 | 0.120 |
| N category | N0/1/2/3 | 10/10/61/31 | 6/7/39/20 | 4/3/22/11 | 0.97 | 0.095 |
| Stage | IIIA/IIIB/IIIC | 62/40/10 | 40/26/6 | 22/14/4 | 0.96 | 0.059 |
| Platinum agent | CBDCA/CDDP | 97/15 | 61/11 | 36/4 | 0.62 | 0.159 |
| Irradiation technique | 3D-CRT/IMRT | 83/29 | 52/20 | 31/9 | 0.70 | 0.122 |
| Treatment volume | IFI/ENI | 43/69 | 24/48 | 19/21 | 0.20 | 0.292 |
| GTV volume | < 57 cm^3^/≥ 57 cm^3^ | 61/51 | 41/31 | 20/20 | 0.61 | 0.140 |
| Hospital volume | High/Low | 83/29 | 56/16 | 27/13 | 0.34 | 0.232 |

*Abbreviations:* SMD, standard mean difference. The other abbreviations are the same as those in Table 1.
